# Asthma is associated with increased severity and duration of rhinitis: A study with the Allergic Rhinitis and its Impact on Asthma classes in the Constances cohort

**DOI:** 10.1002/clt2.12316

**Published:** 2023-11-23

**Authors:** Marine Savouré, Jean Bousquet, Bénédicte Leynaert, Céline Ribet, Marcel Goldberg, Marie Zins, Bénédicte Jacquemin, Rachel Nadif

**Affiliations:** ^1^ Université Paris‐Saclay UVSQ Univ. Paris‐Sud Inserm Equipe d’Epidémiologie Respiratoire Intégrative CESP Villejuif France; ^2^ French Environment and Energy Management Agency Angers France; ^3^ Charité Universitätsmedizin Berlin Humboldt‐Universität zu Berlin Berlin Germany; ^4^ Department of Dermatology and Allergy Comprehensive Allergy Center Berlin Institute of Health Berlin Germany; ^5^ Centre Hospitalier Universitaire Montpellier France; ^6^ MASK‐air Montpellier France; ^7^ Université Paris‐Cité Université Paris‐Saclay UVSQ, Inserm UMS 11 Cohortes Epidémiologiques en population Villejuif France; ^8^ Univ Rennes Inserm EHESP Irset (Institut de recherche en Santé, environnement et travail) ‐ UMR_S 1085 Rennes France

**Keywords:** ARIA, asthma, duration, rhinitis, severity

## Abstract

**Background:**

Few population‐based studies have described allergic rhinitis (AR) according to the Allergic Rhinitis and its Impact on Asthma (ARIA) classification, and none have assessed the impact of asthma on this classification. Our aims were to 1) describe AR according to four ARIA classes and 2) within each of the four ARIA classes, compare participants with AR alone versus those with AR and asthma.

**Methods:**

Cross‐sectional analyses were performed using data from the 2014 annual follow‐up questionnaire of the French adult population‐based cohort Constances. Current AR was defined by the report of sneezing, runny, or blocked nose in the last 12 months and the report of nasal allergies. Following ARIA recommendations, rhinitis was classified according to its severity (mild or moderate‐severe) and duration (intermittent or persistent). Ever asthma was also defined by a questionnaire.

**Results:**

Among the 4675 participants with AR (57% women, mean age 50.2 ± 12.7 years), 44% were classified as mild/intermittent, 16% mild/persistent, 25% moderate‐severe/intermittent, and 15% moderate‐severe/persistent. Within each of the four ARIA classes, compared to participants with rhinitis alone, participants with rhinitis and asthma had significantly more severe symptoms, more conjunctivitis, a higher mean eosinophil count and more treatments with intra‐nasal corticosteroids and oral antihistamines co‐medication.

**Conclusions:**

This is a paradigm shift study as for the first time this large population‐based study in adults showed that asthma status has a profound effect on the ARIA classification. Rhinitis alone and rhinitis with asthma represent two distinct phenotypes. These results reinforce the need to include asthma status in the ARIA classification and guidelines.

## 1 INTRODUCTION

Allergic rhinitis (AR) is one of the most prevalent diseases in the world.^1^ All countries, ethnicities, and age groups are affected by AR, leading to a global health problem.^2^ In this context, during a World Health Organization workshop in 1999, the Allergic Rhinitis and Its Impact on Asthma (ARIA) initiative was initiated. The first ARIA report published in 2001^3^ as a major document for the classification and management of rhinitis was updated or revised in 2008,^2^ 2010,^4^ 2016^5^ and 2022. The ARIA report included a classification of rhinitis according to its severity (“mild” or “moderate‐severe”) and duration (“intermittent” or “persistent”) which allows classification of rhinitis into four classes: mild/intermittent, mild/persistent, moderate‐severe/intermittent and moderate‐severe/persistent.^2^

The ARIA classification has been validated in clinical practice.^2^, ^6^, ^7^, ^8^ Several population‐based studies have described AR according to its duration^9^, ^10^, ^11^, ^12^ or severity^12^, ^13^, ^14^, ^15^ but none have investigated the duration and severity together. In a previous study based on data from the Constances population‐based cohort, we observed a much higher prevalence of mild AR than in clinical practice,^16^ suggesting the existence of rhinitis profiles with different characteristics in the general population, in particular regarding rhinitis severity. Another gap in the literature concerns whether the co‐occurrence of asthma may impact the characteristics of the different AR classes: even if it is known that asthma and AR often coexist, no study has investigated the impact of asthma in the ARIA classification. Furthermore, a recent hypothesis proposed that rhinitis alone is a disease distinct from rhinitis with asthma.^17^ Overall, no population‐based study has been conducted in adults to describe AR according to its severity and duration or to assess if within each of the four ARIA class participants with AR alone are different from those with AR and asthma.

Our aim was to describe AR according to its severity and duration defined by the ARIA classification among adults from the general population. Our specific objectives were to 1) describe AR according to four ARIA classes and 2) within each of the four ARIA classes, compare participants with AR alone versus those with AR and asthma.

## 2 METHODS

### 2.1 Study design

A cross‐sectional study was carried out with the data from the 2014 annual follow‐up questionnaire of the Constances cohort among participants reporting current AR. We first describe AR according to the two major criteria of ARIA that is, severity and duration and then we studied the impact of asthma on these ARIA classes concerning clinical features, eosinophils and medication needs.

### 2.2 Settings and participants

Constances is a population‐based cohort of 220,000 adults aged 18–69 at inclusion, randomly selected from social security affiliates in France (https://www.constances.fr/index_EN.php). The participants were enrolled from 2012 to 2020 in 20 administrative districts. At inclusion, participants completed several standardized questionnaires and had a complete medical examination.^18^, ^19^, ^20^ An annual follow‐up was done through a postal or online self‐questionnaire. The annual follow‐up questionnaires present different questions each year, according to specific research themes. One of the themes of the 2014 annual follow‐up questionnaire was rhinitis. All participants who were included until the end of 2013 received the 2014 annual follow‐up questionnaire, which included two pages of detailed, validated, and standardized questions on rhinitis (online supplement).

All confidentiality, safety and security procedures were approved by the French legal authorities. Approvals were obtained from the National Data Protection Authority on March 3, 2011 (Commission Nationale de l’Informatique et des Libertés—CNIL, French National Data Protection Authority (authorisation no. 910486)), the National Council for Statistical Information (Conseil National de l’Information Statistique—CNIS), the National Medical Council (Conseil National de l’Ordre des Médecins—CNOM), and the Institutional Review Board of the National Institute for Medical Research‐INSERM (authorisation no. 01–011). All participants signed a written informed consent.

### 2.3 AR and asthma

As specific Immunoglobulin E (IgE) and Skin Prick Tests (SPTs) are not available in Constances, we used a definition of AR based on a questionnaire, as published previously.^16^, ^21^ Participants were considered as having current AR if they answered “yes” to all the three following questions: “*During your lifetime, have you ever had a problem with sneezing, or a runny, or a blocked nose when you did not have a cold or the flu?*”, to “*Have you had these problems in the last* 12 months*?”*, and to “*Have you ever had nasal allergies in your lifetime, including hay fever?*”.

Following the ARIA recommendations,^2^ AR was defined as moderate‐severe if at least one of the symptoms of rhinitis: rhinorrhea, nasal congestion, nasal pruritus, or sneezing has been reported as a disturbing problem affecting daily activities and sleep. Otherwise, rhinitis was defined as mild if none of the symptoms have been reported as a disturbing problem affecting daily activities and sleep.

Allergic rhinitis was defined as persistent if symptoms occurred more than 4 days per week and more than four consecutive weeks. Otherwise, AR was defined as intermittent.

Participants were considered as having ever‐asthma if they answered yes to “*Have you ever had asthma?”* at inclusion or answered “asthma” to: “*Here is a list of health problems. Indicate here the ones you have suffered from in the last* 12 months *(whether or not there was a work interruption, whether or not there is a treatment)”* at the 2014 follow‐up questionnaire.

Other variables of interest are described in the supplement.

### 2.4 Statistical analyses

Analysis in complete‐cases, that is, by excluding participants with missing data, was carried out, and no imputation was performed.

We described AR according to the ARIA classification of severity (mild versus moderate‐severe) and duration (intermittent versus persistent) separately. The severity and duration of rhinitis were then combined into four classes according to the ARIA classification: mild/intermittent, mild/persistent, moderate‐severe/intermittent, and moderate‐severe/persistent. Then, we compared participants with AR and never asthma versus those with AR and ever asthma within each of the four ARIA classes.

Pearson Chi‐2 tests for categorical variables and Student *t* test/analysis of variance comparison of variances for continuous variables were used. We computed effect size measures.^22^ For categorical variables (Cramer's V coefficient), values of 0.1–0.3 were considered to represent small effect sizes, 0.3–0.5 medium effect and ≥0.5 large effect sizes.^23^ For continuous variables (Cohen's D coefficient), values of 0.2–0.5 were considered to represent small effect sizes, 0.5–0.8 medium effect and ≥0.8 large effect sizes.^24^ We performed additional analyses with multivariate logistic regressions comparing participants with ever asthma to those with never asthma among the different strata of ARIA classification, adjusting for age, gender, smoking, diploma, conjunctivitis and eczema.

All analyses were performed using SAS 9.4 software (SAS Institute).

## 3 RESULTS

### 3.1 Demographic characteristics of the participants

**FIGURE 1:**
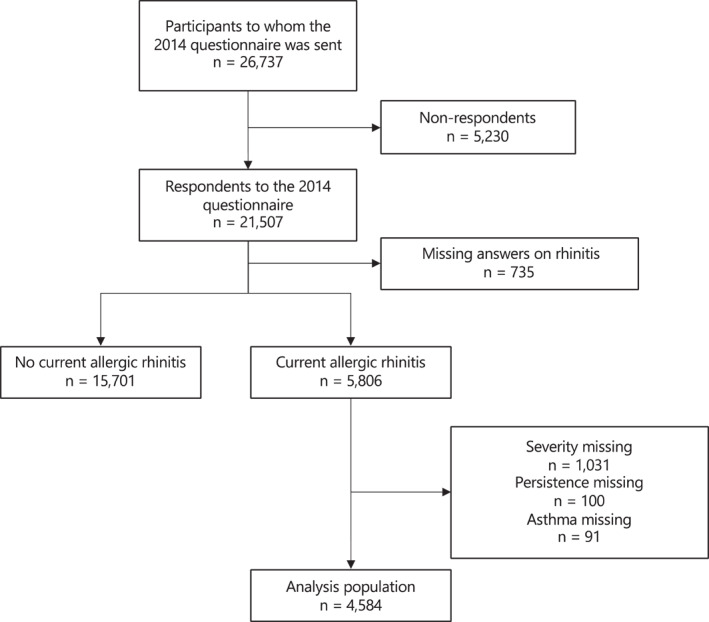
Flow chart. Among the 26,737 participants included in the cohort by 2013, 21,507 (80%) of them completed the 2014 follow‐up questionnaire. Participants with missing data regarding the definition of allergic rhinitis (AR) (*n* = 735) were excluded from the analyses. Among participants with current AR, participants with missing data regarding severity (*n* = 1031), duration (*n* = 100), or ever asthma (*n* = 91) were also excluded. Finally, 4584 participants with AR were included in the present analysis.

Among the 26,737 participants included in the cohort by 2013, 21,507 (80%) of them completed the 2014 follow‐up questionnaire. Participants with missing data regarding the definition of AR (*n* = 735) were excluded from the analyses. Among participants with current AR, participants with missing data regarding severity (*n* = 1031), duration (*n* = 100), or ever asthma (*n* = 91) were also excluded. Finally, 4584 participants with AR were included (Figure 1).

Compared to participants with missing data for severity and duration, participants included in the analyses were younger and had a higher level of education (Table S1).

### 3.2 Descriptive data

Table 1 shows the demographic characteristics of the study population: the mean age was 50.1 years, 57.2% of the participants were women and 25.3% reported ever asthma. Based on the ARIA severity classification, 60.0% of the participants were classified as having mild and 40.0% as having moderate‐severe rhinitis. The mean Total Nasal Symptom Score (TNSS4) was 5.8. Based on the ARIA duration classification, 68.7% of the participants were classified as having intermittent and 31.3% as having persistent rhinitis. By combining the two classifications, 2019 (44.0%) participants were classified as having mild/intermittent, 731 (15.9%) as having mild/persistent, 1131 (24.7%) as having moderate‐severe/intermittent, and 703 (15.3%) as having moderate‐severe/persistent rhinitis.

**TABLE 1:** Demographic characteristics, and allergic rhinitis and its impact on asthma (ARIA) classification of participants with allergic rhinitis (AR).

	Analysis population (*n* = 4584)
Sex, *n* (%)	
Men	1964 (42.8)
Women	2620 (57.2)
Age, years, mean (SD)	50.1 (12.7)
Tobacco status, *n* (%)	
Never smoker	2053 (46.6)
Ex‐smoker	1791 (40.7)
Current smoker	559 (12.7)
Educational level, *n* (%)	
Less than high school	319 (7.0)
High school	1274 (28.0)
University	2957 (65.0)
Asthma, *n* (%)	
Never asthma	3424 (74.7)
Ever asthma	1160 (25.3)
Rhinitis severity, *n* (%)	
Mild	2750 (60.0)
Moderate to severe	1834 (40.0)
TNSS4, mean (SD)	5.8 (2.8)
TNSS4, *n* (%)	
[0–2]	604 (13.2)
[3–4]	1030 (22.5)
[5–6]	1183 (25.8)
≥7	1767 (38.5)
Rhinitis duration, *n* (%)	
Intermittent	3150 (68.7)
Persistent	1434 (31.3)
ARIA classification, *n* (%)	
Mild/Intermittent	2019 (44.0)
Mild/Persistent	731 (15.9)
Moderate‐severe/Intermittent	1131 (24.7)
Moderate‐severe/Persistent	703 (15.3)

*Note*: Data are mean (SD) or *n* (%).

Abbreviation: TNSS4, Total Nasal Symptom Score 4.

### 3.3 AR severity

The characteristics of participants with AR according to its severity are presented in Table 2. There were more women with moderate‐severe AR than with mild AR (62.1% vs. 53.9%, *p* < 0.001). Participants with moderate‐severe AR had a higher mean eosinophil count (218.5 vs. 203.2, *p* = 0.004) and a higher TNSS4 score (7.8 vs. 4.5, *p* < 0.001) as well as an earlier age of onset of rhinitis symptoms (21.4 vs. 24.6, *p* < 0.001) than those with mild AR. Compared to participants with mild AR, those with moderate‐severe AR reported more multi‐morbidities: ever asthma (30.8% vs. 21.6%, <0.0001), ever conjunctivitis (59.7% vs. 51.3%, *p* < 0.0001), and/or ever eczema (44.9% vs. 37.6%, *p* < 0.0001). They also reported more nasal symptoms: rhinorrhea (82.2% vs. 68.6%, *p* < 0.0001), nasal congestion/obstruction (92.2% vs. 63.6%, *p* < 0.0001), nasal itching (71.3% vs. 61.1%, *p* < 0.0001), sneezing (81.1% vs. 69.2%, *p* < 0.0001) and associated‐eye symptoms (73.9% vs. 62.1, *p* < 0.0001). Participants with moderate‐severe AR reported more triggers of rhinitis symptoms: dust mites or house dust (39.3% vs. 32.7%, *p* < 0.0001), animals (15.9% vs. 10.8%, *p* < 0.0001), air pollution (31.1% vs. 24.3%, *p* < 0.0001), change in weather (33.8% vs. 25.2%, *p* < 0.0001), tobacco (8.3% vs. 5.3%, *p* < 0.0001), pollens (56.3% vs. 52.8%, *p* = 0.02), cold air (26.6% vs. 22.9%, *p* = 0.005) or other triggers (14.9% vs. 11.2%, *p* = 0.0002), and more persistent rhinitis (38.3% vs. 26.6%, *p* < 0.0001) than those with mild AR. Regarding treatments, participants with moderate‐severe AR reported more co‐medication (Oral Antihistamines (OA) + Intranasal Corticosteroids (INCS)) than those with mild AR (42.4% vs. 25.7%, *p* < 0.0001). Large effect sizes were observed for TNSS4 as expected, medium effect sizes for the congestion/obstruction symptom and the number of reported symptoms and small effect sizes for asthma, duration, rhinorrhea, nasal itching, sneezing, associated‐eye symptoms, the number of reported triggers and co‐medication.

**TABLE 2:** Characteristics of participants with allergic rhinitis (AR) according to its severity.

	Mild (*n* = 2750)	Mod‐severe (*n* = 1834)	*p*	Effect size
Sex, *n* (%)			<0.0001	0.08
Men	1268 (46.1)	696 (38.0)		
Women	1482 (53.9)	1138 (62.1)		
Age, years, mean (SD)	51.8 (12.5)	47.6 (12.5)	<0.0001	0.34
Tobacco status, *n* (%)			0.08	0.03
Never‐smoker	1220 (46.2)	833 (47.3)		
Ex‐smoker	1106 (41.9)	685 (38.9)		
Current smoker	317 (12.0)	242 (13.8)		
Educational level, *n* (%)			0.64	0.01
Less than high school	183 (6.7)	136 (7.5)		
High school	765 (28.1)	509 (27.9)		
University	1776 (65.2)	1181 (64.7)		
Body‐mass index, kg/m^2^, *n* (%)			0.61	0.02
<18.5	55 (2.0)	45 (2.5)		
[18.5–25]	1602 (59.1)	1037 (57.9)		
[25–30]	780 (28.8)	519 (29.0)		
≥30	272 (10.0)	191 (10.7)		
Asthma, *n* (%)	0 (0.0)		<0.0001	0.10
Never asthma	2155 (78.4)	1269 (69.2)		
Ever asthma	595 (21.6)	565 (30.8)		
Conjunctivitis, *n* (%)			<0.0001	0.08
Never conjunctivitis	1228 (48.7)	686 (40.3)		
Ever conjunctivitis	1292 (51.3)	1017 (59.7)		
Eczema, *n* (%)			<0.0001	0.07
Never eczema	1557 (62.4)	922 (55.1)		
Ever eczema	938 (37.6)	751 (44.9)		
Eosinophils count, cell/mm^3^, mean (SD)	203.2 (139.0)	218.5 (162.3)	0.004	0.10
TNSS4, mean (SD)	4.5 (2.1)	7.8 (2.3)	<0.0001	1.52
Rhinitis duration, *n* (%)			<0.0001	0.12
Intermittent	2019 (73.4)	1131 (61.7)		
Persistent	731 (26.6)	703 (38.3)		
Reported symptoms^a^, *n* (%)	0 (0.0)			
Rhinorrhoea	1885 (68.6)	1508 (82.2)	<0.0001	0.15
Nasal congestion/obstruction	1750 (63.6)	1691 (92.2)	<0.0001	0.32
Nasal itching	1680 (61.1)	1307 (71.3)	<0.0001	0.10
Sneezing	1902 (69.2)	1487 (81.1)	<0.0001	0.13
Associated‐eye symptoms	1693 (62.1)	1348 (73.9)	<0.0001	0.12
Number of reported symptoms, mean (SD)	3.2 (1.3)	4.0 (1.1)	<0.0001	0.63
Age of onset of rhinitis, year, mean (SD)	24.6 (15.5)	21.4 (13.6)	<0.0001	0.21
Reported triggers of symptoms^a^, *n* (%)				
Dust mites or house dust	900 (32.7)	720 (39.3)	<0.0001	0.07
Animals	296 (10.8)	291 (15.9)	<0.0001	0.07
Air pollution	667 (24.3)	571 (31.1)	<0.0001	0.08
Change in weather	692 (25.2)	620 (33.8)	<0.0001	0.09
Tobacco	146 (5.3)	153 (8.3)	<0.0001	0.06
Pollens	1451 (52.8)	1033 (56.3)	0.02	0.04
Cold air	630 (22.9)	487 (26.6)	0.005	0.04
Other	307 (11.2)	274 (14.9)	0.0002	0.06
Unknown	735 (26.7)	459 (25.0)	0.20	0.02
Number of reported triggers, mean (SD)	2.1 (1.2)	2.5 (1.4)	<0.0001	0.31
Rhinitis treatment, *n* (%)			<0.0001	0.21
Neither OA nor INCS	1100 (40.5)	405 (22.4)		0.19
OA only	617 (22.7)	398 (22.0)		0.01
INCS only	303 (11.2)	239 (13.2)		0.04
OA and INCS	698 (25.7)	770 (42.5)		0.18

*Note*: Data are mean (SD) or *n* (%). Orange: Small effect size (Cramer's V from [0.1–0.3] or Cohen's D from [0.2–0.5]); Yellow: medium effect size (Cramer's V from [0.3–0.5] or Cohen's D from [0.5–0.8]); Green: large effect size (Cramer's V ≥ 0.5 or Cohen's *D* ≥ 0.8).

Abbreviations: BMI, Body Mass Index; INCS, Intranasal Corticosteroids; OA, Oral Antihistamines; TNSS4, Total Nasal Symptom Score 4.

^a^ Several possible answers.

### 3.4 AR duration

The characteristics of participants with AR according to its duration are presented in Table 3. No significant differences were observed for gender, age, smoking status, education level and body mass index between groups. Participants with persistent AR had a higher mean eosinophil count (222.1 vs. 203.4, *p* = 0.001), a higher TNSS4 (6.4 vs. 5.6, *p* < 0.0001) and reported more moderate‐severe rhinitis (49.0% vs. 35.9%, *p* < 0.0001) than those with intermittent AR. Participants with persistent AR also reported more ever asthma (28.3% vs. 23.9%, *p* = 0.002) and ever conjunctivitis (58.2% vs. 53.0%, *p* = 0.002). Regarding treatment, participants with persistent AR reported more co‐medication (OA + INCS) than those with intermittent AR (42.6% vs. 27.8%, *p* < 0.0001). Small effect sizes were observed for TNSS4, severity, and co‐medication.

**TABLE 3:** Characteristics of participants with allergic rhinitis (AR) according to its duration.

	Intermittent (*n* = 3150)	Persistent (*n* = 1434)	*p*	Effect size
Sex, *n* (%)			0.10	0.02
Men	1375 (43.7)	589 (41.1)		
Women	1775 (56.4)	845 (58.9)		
Age, years, mean (SD)	50.3 (12.5)	49.8 (13.0)	0.28	0.03
Tobacco status, *n* (%)			0.35	0.02
Never‐smoker	1415 (46.6)	638 (46.8)		
Ex‐smoker	1224 (40.3)	567 (41.6)		
Current smoker	400 (13.2)	159 (11.7)		
Educational level, *n* (%)			0.46	0.02
Less than high school	217 (7.0)	102 (7.2)		
High school	892 (28.6)	382 (26.8)		
University	2014 (64.5)	943 (66.1)		
Body‐mass index, kg/m^2^, *n* (%)			0.07	0.04
<18.5	58 (1.9)	42 (3.0)		
[18.5–25]	1805 (58.3)	834 (59.4)		
[25–30]	905 (29.2)	394 (28.0)		
≥30	328 (10.6)	135 (9.6)		
Asthma, *n* (%)			0.002	0.05
Never asthma	2396 (76.1)	1028 (71.7)		
Ever asthma	754 (23.9)	406 (28.3)		
Conjunctivitis, *n* (%)			0.002	0.05
Never conjunctivitis	1359 (47.0)	555 (41.8)		
Ever conjunctivitis	1535 (53.0)	774 (58.2)		
Eczema, *n* (%)			0.15	0.02
Never eczema	1721 (60.2)	758 (57.9)		
Ever eczema	1137 (39.8)	552 (42.1)		
Eosinophils count, cell/mm^3^, mean (SD)	203.4 (139.7)	222.1 (166.3)	0.001	0.13
TNSS4, mean (SD)	5.6 (2.7)	6.4 (2.8)	<0.0001	0.29
Severity, *n* (%)			<0.0001	0.12
Mild	2019 (64.1)	731 (51.0)		
Moderate‐severe	1131 (35.9)	703 (49.0)		
Reported symptoms^a^, *n* (%)				
Rhinorrhoea	2289 (72.7)	1104 (77.0)	0.002	0.05
Nasal congestion/obstruction	2299 (73.0)	1142 (79.6)	<0.0001	0.07
Nasal itching	2030 (64.4)	957 (66.7)	0.13	0.02
Sneezing	2301 (73.1)	1088 (75.9)	0.04	0.03
Associated‐eye symptoms	2066 (66.1)	975 (68.7)	0.08	0.03
Number of reported symptoms, mean (SD)	3.5 (1.3)	3.7 (1.3)	<0.0001	0.15
Age of onset of rhinitis, year, mean (SD)	23.0 (14.6)	23.7 (15.2)	0.20	0.05
Reported triggers of symptoms^a^, *n* (%)				
Dust mites or house dust	1095 (34.8)	525 (36.6)	0.22	0.02
Animals	401 (12.7)	186 (13.0)	0.82	0.003
Air pollution	790 (25.1)	448 (31.2)	<0.0001	0.06
Change in weather	937 (29.8)	375 (26.2)	0.01	0.04
Tobacco	180 (5.7)	119 (8.3)	0.001	0.05
Pollens	1691 (53.7)	793 (55.3)	0.31	0.02
Cold air	774 (24.6)	343 (23.9)	0.63	0.01
Other	378 (12.0)	203 (14.2)	0.04	0.03
Unknown	757 (24.0)	437 (30.5)	<0.0001	0.07
Number of reported triggers, mean (SD)	2.2 (1.2)	2.4 (1.5)	0.0001	0.13
Rhinitis treatment, *n* (%)			<0.0001	0.17
Neither OA nor INCS	1179 (37.8)	326 (23.1)		0.15
OA only	722 (23.2)	293 (20.7)		0.03
INCS only	350 (11.2)	192 (13.6)		0.03
OA and INCS	865 (27.8)	603 (42.6)		0.15

*Note:* Data are mean (SD) or *n* (%). Orange: small effect size (Cramer's V from [0.1–0.3] or Cohen's D from [0.2–0.5]); Yellow: medium effect size (Cramer's V from [0.3–0.5] or Cohen's D from [0.5–0.8]); Green: large effect size (Cramer's V ≥ 0.5 or Cohen's *D* ≥ 0.8).

Abbreviations: BMI, Body Mass Index; INCS, Intranasal Corticosteroids; OA, Oral Antihistamines; TNSS4, Total Nasal Symptom Score 4.

^a^ Several possible answers.

### 3.5 AR according to the four classes ARIA classification

The characteristics of participants with AR according to the four ARIA classes are presented in Table 4. The four classes had different characteristics: participants with mild AR, whether it was intermittent or persistent, had the lowest prevalences for ever asthma (21.2%), ever conjunctivitis (50.6%), and ever eczema (37.4%), the highest proportion of participants with neither OA nor INCS (43.7%), and the lowest mean eosinophil count (201 ± 138). In contrast, participants with moderate‐severe/persistent AR had the highest prevalences for ever asthma (34.1%), ever conjunctivitis (63.4%), and ever eczema (46.3%), the highest proportion of participants with co‐medication (OA + INCS) (52.3%), and the highest mean eosinophil count (235 ± 188). Participants with mild/persistent rhinitis had the latest age of onset of symptoms and a higher percentage of participants reported not knowing what triggered their symptoms. In contrast, participants with moderate‐severe/persistent rhinitis had the earliest age of onset of symptoms and a lower percentage of participants reporting not knowing what triggered their symptoms.

**TABLE 4:** Characteristics of allergic rhinitis (AR) according to the allergic rhinitis and its impact on asthma (ARIA) classification.

	Mild	Mild	Mod‐severe	Mod‐severe	*p*
	Intermittent	Persistent	Intermittent	Persistent	
	(*n* = 2019)	(*n* = 731)	(*n* = 1131)	(*n* = 703)	
Sex, *n* (%)					<0.0001
Men	937 (46.4)	331 (45.3)	438 (38.7)	258 (36.7)	
Women	1082 (53.6)	400 (54.7)	693 (61.3)	445 (63.3)	
Age, years, mean (SD)	51.9 (12.3)	51.8 (13.2)	47.4 (12.4)	47.8 (12.6)	<0.0001
Tobacco status, *n* (%)					0.06
Never smoker	889 (45.7)	331 (47.6)	526 (48.2)	307 (46.0)	
Ex‐smoker	821 (42.2)	285 (40.9)	403 (36.9)	282 (42.2)	
Current smoker	237 (12.2)	80 (11.5)	163 (14.9)	79 (11.8)	
Educational level, *n* (%)					0.76
Less than high school	134 (6.7)	49 (6.7)	83 (7.4)	53 (7.6)	
High school	564 (28.2)	201 (27.7)	328 (29.2)	181 (25.8)	
University	1300 (65.1)	476 (65.6)	714 (63.5)	467 (66.6)	
BMI, kg/m^2^, *n* (%)					0.36
<18.5	34 (1.7)	21 (2.9)	24 (2.2)	21 (3.1)	
[18.5–25]	1175 (59.1)	427 (59.3)	630 (56.9)	407 (59.4)	
[25–30]	572 (28.8)	208 (28.9)	333 (30.1)	186 (27.2)	
≥30	208 (10.5)	64 (8.9)	120 (10.8)	71 (10.4)	
Asthma, *n* (%)					<0.0001
Never asthma	1590 (78.8)	565 (77.3)	806 (71.3)	463 (65.9)	
Ever asthma	429 (21.2)	166 (22.7)	325 (28.7)	240 (34.1)	
Conjunctivitis, *n* (%)					<0.0001
Never conjunctivitis	913 (49.4)	315 (46.8)	446 (42.6)	240 (36.6)	
Ever conjunctivitis	934 (50.6)	358 (53.2)	601 (57.4)	416 (63.4)	
Eczema, *n* (%)					<0.0001
Never eczema	1144 (62.6)	413 (61.9)	577 (56.0)	345 (53.7)	
Ever eczema	684 (37.4)	254 (38.1)	453 (44.0)	298 (46.3)	
Eosinophil count, cells/mm^3^, mean (SD)	200.7 (138.0)	209.9 (141.3)	208.1 (142.7)	234.7 (188.0)	<0.0001
TNSS4, mean (SD)	4.3 (2.1)	4.8 (2.2)	7.7 (2.3)	8.0 (2.5)	<0.0001
Reported symptoms^a^, *n* (%)					
Rhinorrhoea	1346 (66.7)	539 (73.7)	943 (83.4)	565 (80.4)	<0.0001
Nasal congestion/obstruction	1263 (62.6)	487 (66.6)	1036 (91.6)	655 (93.2)	<0.0001
Nasal itching	1240 (61.4)	440 (60.2)	790 (69.8)	517 (73.5)	<0.0001
Sneezing	1387 (68.7)	515 (70.5)	914 (80.8)	573 (81.5)	<0.0001
Associated‐eye symptoms	1241 (61.9)	452 (62.7)	825 (73.4)	523 (74.8)	<0.0001
Number of reported symptoms, mean (SD)	3.2 (1.3)	3.3 (1.3)	4.0 (1.0)	4.0 (1.1)	<0.0001
Age of onset of rhinitis, year, mean (SD)	24.2 (15.3)	25.4 (16.0)	21.1 (13.3)	22.0 (14.2)	<0.0001
Reported triggers of rhinitis symptoms^a^, *n* (%)					
Dust mites or house dust	654 (32.4)	246 (33.7)	441 (39.0)	279 (39.7)	0.0001
Animals	222 (11.0)	74 (10.1)	179 (15.8)	112 (15.9)	<0.0001
Air pollution	464 (23.0)	203 (27.8)	326 (28.8)	245 (34.9)	<0.0001
Change in weather	541 (26.8)	151 (20.7)	396 (35.0)	224 (31.9)	<0.0001
Tobacco	94 (4.7)	52 (7.1)	86 (7.6)	67 (9.5)	<0.0001
Pollens	1077 (53.3)	374 (51.2)	614 (54.3)	419 (59.6)	0.009
Cold air	468 (23.2)	162 (22.2)	306 (27.1)	181 (25.7)	0.03
Other	213 (10.5)	94 (12.9)	165 (14.6)	109 (15.5)	0.0007
Unknown	497 (24.6)	238 (32.6)	260 (23.0)	199 (28.3)	<0.0001
Number of reported triggers, mean (SD)	2.1 (1.1)	2.2 (1.3)	2.5 (1.3)	2.6 (1.5)	<0.0001
Rhinitis treatment, *n* (%)					<0.0001
Neither OA nor INCS	873 (43.7)	227 (31.5)	306 (27.4)	99 (14.3)	
OA only	456 (22.8)	161 (22.4)	266 (23.8)	132 (19.0)	
INCS only	211 (10.6)	92 (12.8)	139 (12.4)	100 (14.4)	
OA and INCS	458 (22.9)	240 (33.3)	407 (36.4)	363 (52.3)	

*Note*: Data are mean (SD) or *n* (%).

Abbreviations: BMI, Body Mass Index; INCS, Intranasal Corticosteroids; OA, Oral Antihistamines; TNSS4, Total Nasal Symptom Score 4.

^a^ Several possible answers.

### 3.6 Influence of asthma on the ARIA classification

Eight sub‐classes were defined according to the ARIA classification and the ever asthma status in each subclass (Table 5), with the number of participants per subclass ranging from 166 (mild/persistent AR with ever asthma) to 1590 (mild/intermittent AR without ever asthma).

**TABLE 5:** Characteristics of allergic rhinitis (AR) according to the allergic rhinitis and its impact on asthma (ARIA) classification and asthma status.

	Mild intermittent			Mild persistent			Moderate‐severe intermittent			Moderate‐severe persistent		
	Never asthma (*n* = 1590)	Ever asthma (*n* = 429)	ES	Never asthma (*n* = 565)	Ever asthma (*n* = 166)	ES	Never asthma (*n* = 496)	Ever asthma (*n* = 197)	ES	Never asthma (*n* = 463)	Ever asthma (*n* = 240)	ES
Sex, *n* (%)			0.005			0.04			0.01			0.06
Men	736 (46.3)	201 (46.9)		262 (46.4)	69 (41.6)		310 (38.5)	128 (39.4)		179 (38.7)	79 (32.9)	
Women	854 (53.7)	228 (53.2)		303 (53.6)	97 (58.4)		496 (61.5)	197 (60.6)		284 (61.3)	161 (67.1)	
Age, years, mean (SD)	52.6 (12.1)	49.3 (12.7)	0.27**	52.5 (13.2)	49.2 (12.9)	0.26*	48.1 (12.5)	45.7 (12.2)	0.20*	49.0 (12.9)	45.5 (11.7)	0.28*
Tobacco status, *n* (%)			0.03			0.09			0.03			0.03
Never‐smoker	692 (45.1)	197 (47.9)		268 (50.0)	63 (39.4)		375 (48.2)	151 (48.1)		205 (46.8)	102 (44.4)	
Ex‐smoker	657 (42.8)	164 (39.9)		211 (39.4)	74 (46.3)		291 (37.4)	112 (35.7)		183 (41.8)	99 (43.0)	
Current smoker	187 (12.2)	50 (12.2)		57 (10.6)	23 (14.4)		112 (14.4)	51 (16.2)		50 (11.4)	29 (12.6)	
Educational level, *n* (%)			0.05			0.03			0.02			0.04
Less than high school	115 (7.3)	19 (4.5)		40 (7.1)	9 (5.5)		62 (7.7)	21 (6.5)		36 (7.8)	17 (7.1)	
High school	446 (28.3)	118 (27.9)		156 (27.8)	45 (27.4)		231 (28.8)	97 (29.9)		124 (26.9)	57 (23.8)	
University	1014 (64.4)	286 (67.6)		366 (65.1)	110 (67.1)		508 (63.4)	206 (63.6)		301 (65.3)	166 (69.2)	
Body‐mass index, kg/m^2^, *n* (%)			0.03			0.04			0.06			0.05
<18.5	25 (1.6)	9 (2.1)		18 (3.2)	3 (1.8)		20 (2.6)	4 (1.2)		14 (3.1)	7 (3.0)	
[18.5–25]	926 (59.1)	249 (58.9)		329 (59.3)	98 (59.4)		456 (58.1)	174 (54.0)		276 (60.9)	131 (56.5)	
[25–30]	457 (29.2)	115 (27.2)		160 (28.8)	48 (29.1)		230 (29.3)	103 (32.0)		117 (25.8)	69 (29.7)	
≥30	158 (10.1)	50 (11.8)		48 (8.7)	16 (9.7)		79 (10.1)	41 (12.7)		46 (10.2)	25 (10.8)	
Conjunctivitis, *n* (%)			0.15**			0.16**			0.10*			0.17**
Never conjunctivitis	771 (53.5)	142 (35.0)		264 (51.1)	51 (32.7)		340 (45.9)	106 (34.6)		184 (42.6)	56 (25.0)	
Ever conjunctivitis	670 (46.5)	264 (65.0)		253 (48.9)	105 (67.3)		401 (54.1)	200 (65.4)		248 (57.4)	168 (75.0)	
Eczema, *n* (%)			0.09**			0.09*			0.04			0.10*
Never eczema	931 (64.9)	213 (54.1)		328 (64.3)	85 (54.1)		419 (57.4)	158 (52.7)		246 (57.3)	99 (46.3)	
Ever eczema	503 (35.1)	181 (45.9)		182 (35.7)	72 (45.9)		311 (42.6)	142 (47.3)		183 (42.7)	115 (53.7)	
Eosinophils count, cell/mm^3^, mean (SD)	192.4 (133.1)	231.0 (151.1)	0.28**	192.4 (121.4)	268.5 (182.5)	0.55**	192.7 (133.3)	247.1 (157.8)	0.39**	203.0 (155.2)	291.1 (225.0)	0.48*
TNSS4, mean (SD)	4.2 (2.1)	4.8 (2.1)	0.27**	4.6 (2.2)	5.3 (2.1)	0.29*	7.6 (2.2)	7.9 (2.3)	0.14*	7.7 (2.5)	8.5 (2.3)	0.30*
Reported symptoms^a^, *n* (%)												
Rhinorrhoea	1045 (65.7)	301 (70.2)	0.04	411 (72.7)	128 (77.1)	0.04	667 (82.8)	276 (84.9)	0.03	362 (78.2)	203 (84.6)	0.08
Nasal congestion/obstruction	968 (60.9)	295 (68.8)	0.07*	358 (63.4)	129 (77.7)	0.13*	739 (91.7)	297 (91.4)	0.005	428 (92.4)	227 (94.6)	0.04
Nasal itching	945 (59.4)	295 (68.8)	0.08*	332 (58.8)	108 (65.1)	0.05	555 (68.9)	235 (72.3)	0.03	324 (70.0)	193 (80.4)	0.11**
Sneezing	1086 (68.3)	301 (70.2)	0.02	392 (69.4)	123 (74.1)	0.04	638 (79.2)	276 (84.9)	0.07*	359 (77.5)	214 (89.2)	0.14**
Associated‐eye symptoms	932 (59.1)	309 (72.5)	0.11**	346 (62.1)	106 (64.6)	0.02	570 (71.0)	255 (79.4)	0.09*	325 (70.7)	198 (82.9)	0.13**
Number of reported symptoms, mean (SD)	3.1 (1.3)	3.5 (1.3)	0.28**	3.3 (1.3)	3.6 (1.2)	0.24*	3.9 (1.1)	4.1 (1.0)	0.18*	3.9 (1.2)	4.3 (1.0)	0.39**
Age of onset of rhinitis, year, mean (SD)	26.0 (15.4)	18.6 (13.5)	0.49**	27.5 (16.0)	18.7 (14.3)	0.57**	23.2 (13.4)	16.1 (11.6)	0.56**	24.4 (14.0)	17.8 (13.4)	0.48**
Reported triggers of symptoms^a^, *n* (%)												
Dust mites or house dust	412 (25.9)	242 (56.4)	0.27**	165 (29.2)	81 (48.8)	0.17**	275 (34.1)	166 (51.1)	0.16**	137 (29.6)	142 (59.2)	0.29**
Animals	120 (7.6)	102 (23.8)	0.21**	38 (6.7)	36 (21.7)	0.21**	86 (10.7)	93 (28.6)	0.22**	49 (10.6)	63 (26.3)	0.20**
Air pollution	339 (21.3)	125 (29.1)	0.08*	153 (27.1)	50 (30.1)	0.03	223 (27.7)	103 (31.7)	0.04	153 (33.1)	92 (38.3)	0.05
Change in weather	412 (25.9)	129 (30.1)	0.04	115 (20.4)	36 (21.7)	0.01	273 (33.9)	123 (37.9)	0.04	144 (31.1)	80 (33.3)	0.02
Tobacco	65 (4.1)	29 (6.8)	0.05*	29 (5.1)	23 (13.9)	0.14*	46 (5.7)	40 (12.3)	0.11*	37 (8.0)	30 (12.5)	0.07*
Pollens	816 (51.3)	261 (60.8)	0.08*	267 (47.3)	107 (64.5)	0.14**	409 (50.7)	205 (63.1)	0.11*	249 (53.8)	170 (70.8)	0.16**
Cold air	358 (22.5)	110 (25.6)	0.03	130 (23.0)	32 (19.3)	0.04	212 (26.3)	94 (28.9)	0.03	111 (24.0)	70 (29.2)	0.06
Other	180 (11.3)	33 (7.7)	0.05*	62 (11.0)	32 (19.3)	0.10*	117 (14.5)	48 (14.8)	0.003	66 (14.3)	43 (17.9)	0.05
Unknown	432 (27.2)	65 (15.2)	0.11**	204 (36.1)	34 (20.5)	0.14*	205 (25.4)	55 (16.9)	0.09*	162 (35.0)	37 (15.4)	0.21**
Number of reported triggers, mean (SD)	2.0 (1.1)	2.6 (1.3)	0.52**	2.1 (1.2)	2.6 (1.6)	0.41**	2.3 (1.2)	2.9 (1.5)	0.43**	2.4 (1.5)	3.0 (1.6)	0.42**
Rhinitis treatment, *n* (%)			0.22**			0.21**			0.20**			0.20**
Neither OA nor INCS	758 (48.1)	115 (27.3)	0.17	198 (35.6)	29 (17.7)	0.16	251 (31.5)	55 (17.1)	0.15	84 (18.4)	15 (6.3)	0.16
OA only	340 (21.6)	116 (27.5)	0.06	123 (22.1)	38 (23.2)	0.01	188 (23.6)	78 (24.3)	0.01	91 (19.9)	41 (17.3)	0.03
INCS only	181 (11.5)	30 (7.1)	0.06	77 (13.9)	15 (9.2)	0.06	112 (14.1)	27 (8.4)	0.08	71 (15.5)	29 (12.2)	0.04
OA and INCS	297 (18.9)	161 (38.2)	0.19	158 (28.4)	82 (50.0)	0.19	246 (30.9)	161 (50.2)	0.18	211 (46.2)	152 (64.1)	0.17

*Note*: Data are mean (SD) or *n* (%). Orange: Small effect size (Cramer's V from [0.1–0.3] or Cohen's D from [0.2–0.5]); Yellow: Medium effect size (Cramer's V from [0.3–0.5] or Cohen's D from [0.5–0.8]); Green: Large effect size (Cramer's V ≥ 0.5 or Cohen's *D* ≥ 0.8).

Abbreviations: BMI, Body Mass Index; INCS, Intranasal Corticosteroids; OA, Oral Antihistamines; TNSS4, Total Nasal Symptom Score 4.

^a^ Several possible answers.

*p < 0.05; **p < 0.0001.

Whatever the ARIA subclass considered, within each ARIA subclass, asthma multimorbidity further separated participants, showing significant differences for most outcomes. In particular, ever conjunctivitis was higher by 10%–20% in AR and ever asthma in comparison to AR alone for all 4 ARIA classes (small effect size). In each category, the largest effect size between participants without ever asthma and with ever asthma was for the age of onset of rhinitis. The age of onset of rhinitis ranged from 16.1 years (moderate‐severe/intermittent AR + ever asthma) to 26.0 years (mild/intermittent AR alone). Importantly, co‐medication (OA + INCS) was reported from 18.8% (mild intermittent AR alone) to 64.1% (moderate‐severe AR + ever asthma). The prevalence of reported symptoms was always higher in the AR + ever asthma group regardless of the ARIA class. Reported triggers of rhinitis symptoms were far higher in AR + ever asthma than AR alone for mites or house dust and animals. Eosinophil counts were significantly higher for all ARIA classes in participants with asthma than in those with rhinitis alone (small to medium effect sizes). Of note, mean eosinophil counts varied little across the ARIA classes in participants without asthma (range from 192 to 203 cells/mm^3^), but more marked differences between classes were observed in participants with ever asthma (range from 231 to 291 cells/mm^3^). The results of the multivariate logistic regressions after adjustment for age, gender, smoking, diploma, conjunctivitis and eczema are presented in the Table S2. Overall, the strength of the effects and the significance were the same as in the unadjusted analyses.

## 4 DISCUSSION

For the first time in a population‐based study in adults, AR has been described according to the four classes of ARIA classification. More symptoms, conjunctivitis, and eczema were associated with moderate‐severe AR. Asthma prevalence, treatments and reports of rhinitis triggers were higher for participants with moderate‐severe and/or persistent rhinitis. Considering asthma status provided additional information to the ARIA classification. The major finding is that within each of the four ARIA classes, compared to participants with rhinitis alone, participants with rhinitis and asthma had significantly more severe nasal symptoms, more conjunctivitis, higher mean eosinophil count, and more need for INCS and OA co‐medication.

### 4.1 Strengths and limitations

Constances is the largest French population‐based epidemiological study in adults, presenting in 2014 a detailed questionnaire on rhinitis which allowed us to describe in detail the characteristics of AR according to the ARIA classification. Constances is however not fully representative of the French adult population as 1) participants were randomly selected from the affiliated of the National Health Insurance Fund (“Caisse nationale d'assurance maladie”, CNAM). In France, the General Health Insurance Fund administered by CNAM is compulsory for salaried workers, even during unemployed periods and their families; it covers about 85% of the population living in France. Individuals not covered by this scheme are those belonging to the agricultural scheme, which provides social protection for farmers and agricultural workers, the schemes for the liberal professions, and special schemes (such as those for the French National Railway Company). As a result, some of the workers associated with these professions could not be included in Constances. 2) some geographical areas of France were not included. In addition, about 20% of participants had missing data for rhinitis severity. Compared to the participants included in the analysis population, the participants with missing data had a lower level of education and a higher average age, and we cannot exclude that they may have a particular rhinitis profile.

As specific IgE and SPTs are not available in Constances, we used a definition of AR based on a questionnaire. We acknowledge that defining AR by questionnaire may be considered a limitation, even if we have used validated and standardized questions.^25^ We recently showed that the definition we have used is a suitable proxy for AR,^21^ and we found the known characteristics of AR using this definition in Constances.^16^ These questions have the advantage of being based on the main symptoms of AR and are understandable to all participants, which allows all rhinitis to be considered, even those that have not been diagnosed by a doctor. This is especially important as many patients with mild symptoms do not consult a doctor for their rhinitis. Although the questionnaire‐based definition of asthma could be perceived as a limitation, it is important to note that many previous epidemiological studies have already used and validated our definitions based on the European community respiratory health survey questionnaire.^25^, ^26^

### 4.2 Interpretation

1. The percentage of patients with moderate‐severe rhinitis was lower in Constances than in the patient cohorts both in primary^27^ and specialist care.^7^, ^28^, ^29^, ^30^ This is not surprising as it is known that patients who consult a health professional are mainly those with severe symptoms. Few population‐based studies have estimated the prevalence of rhinitis according to its severity. Participants with moderate‐severe AR reported a higher TNSS4 score as compared to those with mild AR, as previously reported in the literature.^8^, ^31^ Similar to Antonicelli *et al.*,^32^ in our study, the participants with mild rhinitis reported less treatment than those with moderate‐severe rhinitis. In four previous studies,^12^, ^13^, ^14^, ^15^ the prevalence of moderate to severe rhinitis ranged from 56% to 87%, which is higher than the prevalence observed in our study; however, the differences in AR definitions across studies make comparisons difficult.
2. Severity impacts more the characteristics of AR than duration, as shown by the higher effect sizes observed for severity than for duration. This was already observed in patients consulting for rhinitis in primary care.^27^ Thus, (i) the treatment strategy should be based on severity. This is reflected by real‐world data obtained from an app showing that European patients treat themselves according to symptoms^33^ (ii) Duration is important for its association with asthma and to give an indication to the physician for the duration of the treatment.
3. The ARIA classification has been criticized for only considering the severity of rhinitis in a dichotomous way: indeed, the large prevalence of moderate‐severe rhinitis found in patient cohorts suggests an important heterogeneity in this disease severity group.^31^ Noteworthy, in Constances, we observed that participants with mild AR constituted more than half of the participants with AR. As an alternative to the classification into two classes, a three‐class classification has been proposed (mild, moderate and severe).^34^ However, we were unable to carry out analyses using this classification, given the way the questions were asked in the Constances.
4. The major novel finding is the impact of asthma on rhinitis in all four ARIA classes. For most important outcomes, asthma has a significant impact (conjunctivitis, eczema, eosinophil counts or combined treatment). The effect size was often moderate, except for treatment (INCS + OA) and the age of onset where it was stronger, and the results persisted after adjusting for age, gender, smoking, diploma, conjunctivitis and eczema. An extremely important data is the treatments reported. For oral anti‐histamines, asthma does not make any difference. More patients without asthma reported intra‐nasal corticosteroids. On the other hand, there was a major impact on asthma by increasing the use of combined intra‐nasal corticosteroid and oral anti‐histamine by around 20% for the four ARIA classes. It has been found in real‐world data of over 10,000 patients that patients with rhinitis using this combination are less well controlled than those using intra‐nasal corticosteroids. These findings were observed in both cross‐sectional and longitudinal studies.^35^, ^36^ In the present study, around 65% of patients with moderate‐severe rhinitis and asthma reported this treatment. On the other hand, it was expected from the ARIA‐MeDALL hypothesis that the age of onset of rhinitis and asthma was earlier than for rhinitis alone and was confirmed in the present study. The present study shows for the first time that the ARIA classification needs to be revised taking into account asthma multimorbidity.

In conclusion, for the first time in a large population‐based study in adults, the characteristics of AR according to the four ARIA classes were described. This result confirms what has been observed in clinical practice and highlights the interest of using the ARIA classification to define AR in the general population. Furthermore, asthma status added important information to the ARIA classification. These findings indicate that ARIA or other rhinitis guidelines should make a distinction between rhinitis alone and rhinitis with asthma for AR management. This critical information is already considered in the development of ARIA 2024.

## AUTHOR CONTRIBUTIONS

**Marine Savouré**: Conceptualization, methodology, formal analysis, writing – original draft, visualization. **Jean Bousquet**: Conceptualization, methodology, writing – review & editing. **Bénédicte Leynaert**: Review & editing. **Céline Ribet**: Resources, review & editing. **Marcel Goldberg**: Resources, review & editing. **Marie Zins**: Resources, review & editing. **Bénédicte Jacquemin**: Conceptualization, methodology, writing – review & editing, supervision. **Rachel Nadif**: Conceptualization, methodology, writing – review & editing, supervision.

## CONFLICT OF INTEREST STATEMENT

Dr. Bousquet reports personal fees from Cipla, Menarini, Mylan, Novartis, Purina, Sanofi‐Aventis, Teva, Uriach, other from KYomed‐Innov, and other from Mask‐air‐SAS, outside the submitted work. The other authors have nothing to disclose.

## CLINICAL IMPLICATIONS

Our findings strongly indicate for the first time that rhinitis guidelines should make a distinction between AR alone and AR with asthma for AR management.

## CAPSULE SUMMARY

For the first time in a large population‐based study in adults, AR according to the four AR and Its Impact on Asthma classes was described. Asthma status added important information to the classification.

## Supporting information

Supplementary Material

## Data Availability

The data that support the findings of this study are available from CONSTANCES. Restrictions apply to the availability of these data, which were used under license for this study. Data are available from https://www.constances.fr with the permission of CONSTANCES.

## References

[1] Bousquet J , Anto JM , Bachert C , et al. Allergic rhinitis. Nat Rev Dis Prim. 2020;6(1):95. 10.1038/s41572-020-00227-0 33273461

[2] Bousquet J , Khaltaev N , Cruz AA , et al. Allergic rhinitis and its impact on asthma (ARIA) 2008 update (in collaboration with the world health organization, GA(2)LEN and AllerGen). Allergy. 2008;63(Suppl 86):8‐160. 10.1111/j.1398-9995.2007.01620.x 18331513

[3] Bousquet J , Van Cauwenberge P , Khaltaev N , Aria Workshop Group, World Health Organization . Allergic rhinitis and its impact on asthma. J Allergy Clin Immunol. 2001;108(5):S147‐S334. 10.1067/mai.2001.118891 11707753

[4] Brozek JL , Bousquet J , Baena‐Cagnani CE , et al. Allergic rhinitis and its impact on asthma (ARIA) guidelines: 2010 revision. J Allergy Clin Immunol. 2010;126(3):466‐476. 10.1016/j.jaci.2010.06.047 20816182

[5] Brożek JL , Bousquet J , Agache I , et al. Allergic rhinitis and its impact on asthma (ARIA) guidelines‐2016 revision. J Allergy Clin Immunol. 2017;140:950‐958. 28602936 10.1016/j.jaci.2017.03.050

[6] Demoly P , Allaert F.‐A , Lecasble M , Bousquet J , PRAGMA . Validation of the classification of ARIA (allergic rhinitis and its impact on asthma). Allergy. 2003;58(7):672‐675. 10.1034/j.1398-9995.2003.t01-1-00202.x 12823130

[7] Bousquet J , Annesi‐Maesano I , Carat F , et al. Characteristics of intermittent and persistent allergic rhinitis: DREAMS study group. Clin Exp Allergy. 2005;35(6):728‐732. 10.1111/j.1365-2222.2005.02274.x 15969662

[8] del Cuvillo A , Montoro J , Bartra J , et al. Validation of ARIA duration and severity classifications in Spanish allergic rhinitis patients ‐ the ADRIAL cohort study. Rhinology. 2010;48(2):201‐205. 10.4193/rhin09.099 20502761

[9] Bauchau V , Durham SR . Epidemiological characterization of the intermittent and persistent types of allergic rhinitis. Allergy. 2005;60(3):350‐353. 10.1111/j.1398-9995.2005.00751.x 15679721

[10] Ostovar A , Pordel S , Movahed A , et al. The prevalence of allergic rhinitis in Southwestern Iran and its association with chronic rhinosinusitis: a GA2LEN study. Iran J Allergy, Asthma Immunol. 2021;20:263‐270. 10.18502/ijaai.v20i3.6342 34134447

[11] Todo‐Bom A , Loureiro C , Almeida MM , et al. Epidemiology of rhinitis in Portugal: evaluation of the intermittent and the persistent types. Allergy. 2007;62(9):1038‐1043. 10.1111/j.1398-9995.2007.01448.x 17686106

[12] Bachert C , van Cauwenberge P , Olbrecht J , van Schoor J . Prevalence, classification and perception of allergic and nonallergic rhinitis in Belgium. Allergy. 2006;61(6):693‐698. 10.1111/j.1398-9995.2006.01054.x 16677237

[13] Almehizia AA , AlEssa RK , Alwusaidi KM , et al. Allergic rhinitis: disease characteristics and coping measures in Saudi Arabia. PLoS One. 2019;14(6):e0217182. 10.1371/journal.pone.0217182 31242201 PMC6594581

[14] Al‐Digheari A , Mahboub B , Tarraf H , et al. The clinical burden of allergic rhinitis in five Middle Eastern countries: results of the SNAPSHOT program. Allergy Asthma Clin Immunol. 2018;14(1):63. 10.1186/s13223-018-0298-x 30473712 PMC6240937

[15] Li CW , Chen DH , Zhong JT , et al. Epidemiological characterization and risk factors of allergic rhinitis in the general population in Guangzhou City in China. PLoS One. 2014;9(12):e114950. 10.1371/journal.pone.0114950 25514026 PMC4267734

[16] Savouré M , Bousquet J , Leynaert B , et al. Rhinitis phenotypes and multimorbidities in the general population: the CONSTANCES cohort. Eur Respir J. 2023;61(2):2200943. 10.1183/13993003.00943-2022 36202419 PMC9909208

[17] Bousquet J , Melén E , Haahtela T , et al. Rhinitis associated with asthma is distinct from rhinitis alone: the ARIA‐MeDALL hypothesis. Allergy. 2023;78(5):1169‐1203. 10.1111/all.15679 36799120

[18] Zins M , Goldberg M , CONSTANCES team . The French CONSTANCES population‐based cohort: design, inclusion and follow‐up. Eur J Epidemiol. 2015;30(12):1317‐1328. 10.1007/s10654-015-0096-4 26520638 PMC4690834

[19] Goldberg M , Carton M , Descatha A , et al. CONSTANCES: a general prospective population‐based cohort for occupational and environmental epidemiology: cohort profile. Occup Environ Med. 2017;74(1):66‐71. 10.1136/oemed-2016-103678 27884936 PMC5241503

[20] Henny J , Nadif R , Got SL , et al. The CONSTANCES cohort biobank: an open tool for research in epidemiology and prevention of diseases. Front Public Health. 2020;8:605133. 10.3389/fpubh.2020.605133 33363097 PMC7758208

[21] Savouré M , Bousquet J , Burte E , et al. Questionnaire as an alternative of skin prick tests to differentiate allergic from non‐allergic rhinitis in epidemiological studies. Allergy. 2021;76(7):2291‐2294. 10.1111/all.14812 33711176

[22] Ordak M . Biostatistics in allergy – recommendations for authors. Allergy. 2022;77(12):3493‐3495. 10.1111/all.15463 35916056

[23] Kim H.‐Y . Statistical notes for clinical researchers: Chi‐squared test and Fisher’s exact test. Restor Dent Endod. 2017;42(2):152. 10.5395/rde.2017.42.2.152 28503482 PMC5426219

[24] Cohen J Statistical Power Analysis for the Behavioral Sciences. 0 ed. Routledge; 2013. 10.4324/9780203771587

[25] Variations in the prevalence of respiratory symptoms, self‐reported asthma attacks, and use of asthma medication in the European Community Respiratory Health Survey (ECRHS). Eur Respir J 1996;9:687‐695. 8726932 10.1183/09031936.96.09040687

[26] Nadif R , Savouré M . Chapter 1 ‐ asthma: from one disease to endotypes. In: Nadif R , ed. Asthma in the 21st Century. Academic Press; 2023:1‐30.

[27] Bousquet J , Neukirch F , Bousquet PJ , et al. Severity and impairment of allergic rhinitis in patients consulting in primary care. J Allergy Clin Immunol. 2006;117(1):158‐162. 10.1016/j.jaci.2005.09.047 16387600

[28] Colás C , Galera H , Añibarro B , et al. Disease severity impairs sleep quality in allergic rhinitis (The SOMNIAAR study). Clin Exp Allergy. 2012;42(7):1080‐1087. 10.1111/j.1365-2222.2011.03935.x 22251258

[29] Valero A , Justicia JL , Antón E , et al. Epidemiology of allergic rhinitis caused by grass pollen or house‐dust mites in Spain. Am J Rhinol Allergy. 2011;25(4):e123‐e128. 10.2500/ajra.2011.25.3599 21310119

[30] Braido F , Baiardini I , Scichilone N , et al. Illness perception, mood and coping strategies in allergic rhinitis: are there differences among ARIA classes of severity? Rhinology. 2014;52(1):66‐71. 10.4193/rhin13.040 24618631

[31] Valero A , Ferrer M , Sastre J , et al. A new criterion by which to discriminate between patients with moderate allergic rhinitis and patients with severe allergic rhinitis based on the Allergic Rhinitis and its Impact on Asthma severity items. J Allergy Clin Immunol. 2007;120(2):359‐365. 10.1016/j.jaci.2007.04.006 17531304

[32] Antonicelli L , Micucci C , Voltolini S , et al. Relationship between ARIA classification and drug treatment in allergic rhinitis and asthma. Allergy. 2007;62(9):1064‐1070. 10.1111/j.1398-9995.2007.01470.x 17686109

[33] Sousa‐Pinto B , Sá‐Sousa A , Vieira RJ , et al. Behavioural patterns in allergic rhinitis medication in Europe: a study using MASK‐air® real‐world data. Allergy. 2022;77(9):2699‐2711. 10.1111/all.15275 35258105

[34] Montoro J , Del Cuvillo A , Mullol J , et al. Validation of the modified allergic rhinitis and its impact on asthma (ARIA) severity classification in allergic rhinitis children: the PEDRIAL study. Allergy. 2012;67(11):1437‐1442. 10.1111/all.12011 22985483

[35] Sousa‐Pinto B , Schünemann HJ , Sá‐Sousa A , et al. Comparison of rhinitis treatments using MASK‐air® data and considering the minimal important difference. Allergy. 2022;77(10):3002‐3014. 10.1111/all.15371 35567393

[36] Sousa‐Pinto B , Schünemann HJ , Sá‐Sousa A , et al. Consistent trajectories of rhinitis control and treatment in 16,177 weeks: the MASK‐air® longitudinal study. Allergy. Published Online First. 2022;78(4):968‐983. 10.1111/all.15574 36325824

