# Supplementary material for: Asthma is associated with increased severity and duration of rhinitis: A study with the Allergic Rhinitis and its Impact on Asthma classes in the Constances cohort
Source: Clin Transl Allergy. 2023 Nov 23;13(11):e12316. doi: 10.1002/clt2.12316 (PMC10668004; doi:10.1002/clt2.12316)
Supplement: Supplementary file 1 — Supplementary Material [file CLT2-13-e12316-s001.docx]

# Supporting Information

**Asthma increases the severity and duration of rhinitis: a study with the Allergic Rhinitis and Its Impact on Asthma (ARIA) classes in the Constances cohort** Marine Savouré, Jean Bousquet, Bénédicte Leynaert, Céline Ribet, Marcel Goldberg, Marie Zins, Bénédicte Jacquemin, Rachel Nadif

# Supplement on the method

**Rhinitis questionnaire**

English translation of the rhinitis part of the 2014 annual follow-up questionnaire available in French at: <https://www.constances.fr/S2014>

Q.22: “*During your lifetime, have you ever had any nasal allergies including hay fever?”* Yes/No

Q.23: *“During your lifetime, have you ever had a problem with sneezing, or a runny, or a blocked nose when you did not have a cold or the flu?”* Yes/No

If yes:

- Q23.A: “*Did your eyes itch or cry when you had these nose problems?*” Yes/No
- Q23.B: “*How old were you the first time?*” …. Years
- Q23.C: “*Have you had these nose problems in the last 12 months?*” Yes/No

Questions 24 to 29 ask about nose problems you have had in the last 12 months.

Q.24: “*In which month did you have these nose problems? (several answers possible)*” January/ February/ March/ April/ May/ June/ July/ August/ September/ October/ November/ December

Q.25: “*What factor triggered or increased these nose problems? (several answers possible)”* Dust mites or house dust/ Animals/ Air pollution/ Change in weather/ Tobacco/ Pollens/ Cold air/ Other/ Unknown

Q.26: “*Have you had these nose problems for more than 4 days in a week?”* Yes/No

If yes

- Q.26.A: “*Did these problems last more than 4 consecutive weeks?”* Yes/No

Q27: “*For each of the following problems, indicate whether you have had it in the last 12 months and whether it has been bothersome. If you have not been affected by any of them, indicate this in the first column of the table.”*

|  | I didn’t have this problem | I had this problem but: | | |
| --- | --- | --- | --- | --- |
|  |  | It didn't bother me | It bothered me without affecting my daily activities or my sleep | It has bothered me and affected my daily activities or sleep |
| The nose that flows like water |  |  |  |  |
| Blocked nose (feeling like you can't breathe through your nose) |  |  |  |  |
| Itchy nose |  |  |  |  |
| Sneezing that is particularly violent and occurs in attacks |  |  |  |  |
| Eyes that cry, red, itchy |  |  |  |  |

Q.28: *“Have you used nasal corticosteroid sprays to treat these nose problems? (Nasacort, Nasonex, Avamys, Rhinocort, Beconase, Beclometasone...)”* Yes/No

Q.29: “*Have you used any oral antihistamines/anti-allergic treatments to treat these nose problems? (Aerius, Xyzall, Clarityne, Kestin, Virlix, Zyrtec, Cetirizine, Loratadine, Desloratadine...)”* Yes/No

# Definitions of variables

**Tobacco** **status** was evaluated into three categories: never smoker, ex-smoker and current smoker.

**Educational level** was evaluated at inclusion into three categories: less than high school (no diploma/grade school), high school (baccalauréat or equivalent) or university (≥ baccalauréat +2).

**Body Mass Index** (BMI) was defined as the body mass divided by the square of the body height. Height was measured at the health centre at inclusion and weight was reported by participants in the follow-up questionnaire of 2014.

A White blood (WBC) count was performed at inclusion for each participant in each Health Preventive Centre by an accredited laboratory. Participants were instructed to fast for 12 hours before the blood test which was performed between 8 AM and 10 AM. The WBC results indicate the total count of leukocytes, expressed as 10^9^/L, and the percentage of each of the five leukocyte components: lymphocytes, monocytes, basophils, **eosinophils**, and neutrophils. From the eosinophils and neutrophils, expressed as a percentage of total leukocytes, the numbers of eosinophils and neutrophils, expressed in 10^9^/L were generated (1).

The follow-up questionnaire of 2014 presents detailed questions about rhinitis that defined the following variables:

For **rhinorrhoea, nasal itching, nasal obstruction, sneezing, and associated eye** **symptoms**, participants answered to the question: “*For each of the following problems, indicate whether you have had it in the last 12 months and whether it has been bothersome. If you have not been affected by any of them, indicate this in the first column of the table.*”. Each symptom was scored on a scale from 0 to 3: 0 = no symptom, 1 = a symptom that is present but not bothersome, 2 = a symptom that is bothersome but does not affect daily activities or sleep, and 3 = a symptom that is bothersome and affects daily activities or sleep. Each of the symptoms was considered dichotomously: symptom absent (score = 0) or symptom present (score ≥ 1). The variable “**number of symptoms**” refers to the number of symptoms present (score ≥ 1), considering rhinorrhoea, nasal itching, nasal obstruction, sneezing, and associated eye symptoms (ranges from 0 to 5).

The severity of rhinitis was also considered according to the **total nasal symptom score 4** (TNSS4) which is a score based on the severity of four rhinitis symptoms: rhinorrhea, nasal itching, nasal obstruction and sneezing (2). Each symptom was scored on a scale from 0 to 3: 0 = no symptom, 1 = a symptom that is present but not bothersome, 2 = a symptom that is bothersome but does not affect daily activities or sleep, and 3 = a symptom that is bothersome and affects daily activities or sleep. The overall TNSS4 is the sum of all four symptoms score ranging from 0 to 12.

**Age of onset of rhinitis**: reporting of ever rhinitis and age reported to “*How old were you the first time?”.*

**Triggers** were considered as dichotomous variables (yes/no) depending on whether the participant reported the trigger at the question “*What factor triggered or increased these nose problems? (Several answers possible)”* *Dust mites or house dust, Animals, Air pollution, Change in weather, Tobacco, Pollens, Cold air, Other, Unknown*. The variable “**number of triggers”** refers to the number of triggers reported.

The **treatments of rhinitis** were considered in a four-class variable based on the answers to the questions on oral antihistamines (OA): “*Have you used any oral antihistamines/anti-allergic treatments to treat these nose problems? (Aerius, Xyzall, Clarityne, Kestin, Virlix, Zyrtec, Cetirizine, Loratadine, Desloratadine...)”* and on intranasal corticosteroids (INCS): “*Have you used nasal corticosteroid sprays to treat these nose problems? (Nasacort, Nasonex, Avamys, Rhinocort, Beconase, Beclometasone...)”*. A participant who answered “no” to both questions was categorised as neither OA nor INCS. A participant who answered “yes” to the question on OA and “no” to question on INCS was categorized as OAH only. A participant who answered “no” to question on OA and “yes” to question on INCS was categorised as INCS only. Finally, a participant who answered “yes” to both questions was categorised as OA and INCS.

**Ever conjunctivitis**: yes to *“During your lifetime, have you ever had allergic conjunctivitis?”*

**Ever** **eczema**: yes to *“During your lifetime, have you ever had eczema?”*

Reference:

1. Tsiavia T, Henny J, Goldberg M, Zins M, Roche N, Orsi L, Nadif R. Blood inflammatory phenotypes were associated with distinct clinical expressions of asthma in adults from a large population-based cohort. *eBioMedicine* 2022; 76: 103875.
2. Li AR, Zhang K, Reddy PD, Nguyen SA, Miglani A, Fried J, et al. Systematic review of measures of disease severity in rhinitis. Int Forum Allergy Rhinol. 2021;11:1367–77.

# Tables

Table S1: Comparison of participants included in the analyses with those not included (with missing data for severity or duration or asthma)

|  | Non-included participants  (n=1,222) | Included participants  (n=4,584) |
| --- | --- | --- |
| Sex |  |  |
| Men | 504 (41.2) | 1,964 (42.8) |
| Women | 718 (58.8) | 2,620 (57.2) |
| Age, years |  |  |
| Missing | 0 (0.0) | 0 (0.0) |
| Means (SD) | 57.5 (11.2) | 50.1 (12.7) |
| Tobacco status |  |  |
| Missing | 114 (9.3) | 181 (4.0) |
| Never smoker | 481 (39.4) | 2,053 (44.8) |
| Ex-smoker | 498 (40.8) | 1,791 (39.1) |
| Current smoker | 129 (10.6) | 559 (12.2) |
| Educational level |  |  |
| Missing | 32 (2.6) | 34 (0.7) |
| Less than high school | 176 (14.4) | 319 (7.0) |
| High school | 443 (36.3) | 1,274 (27.8) |
| University | 571 (46.7) | 2,957 (64.5) |
| Body-mass index, kg/m^2^ |  |  |
| Missing | 46 (3.8) | 83 (1.8) |
| <18.5 | 24 (2.0) | 100 (2.2) |
| [18.5 - 25[ | 625 (51.2) | 2,639 (57.6) |
| [25 - 30[ | 391 (32.0) | 1,299 (28.3) |
| ≥30 | 136 (11.1) | 463 (10.1) |
| Asthma |  |  |
| Missing | 119 (9.7) | 0 (0.0) |
| Never asthma | 880 (72.0) | 3,424 (74.7) |
| Ever asthma | 223 (18.3) | 1,160 (25.3) |
| Conjunctivitis |  |  |
| Missing | 260 (21.3) | 361 (7.9) |
| Never conjunctivitis | 403 (33.0) | 1,914 (41.8) |
| Ever conjunctivitis | 559 (45.7) | 2,309 (50.4) |
| Eczema |  |  |
| Missing | 288 (23.6) | 416 (9.1) |
| Never eczema | 542 (44.4) | 2,479 (54.1) |
| Ever eczema | 392 (32.1) | 1,689 (36.9) |
| Eosinophils count, cell/mm^3^ |  |  |
| Missing | 268 (21.9) | 1,037 (22.6) |
| Means (SD) | 206.0 (209.3) | 136.6 (148.9) |
| Rhinitis severity |  |  |
| Missing | 1,031 (84.4) | 0 (0.0) |
| Mild | 112 (9.2) | 2,750 (60.0) |
| Moderate-severe | 79 (6.5) | 1,834 (40.0) |
| TNSS4 |  |  |
| Missing | 1,031 (84.4) | 0 (0.0) |
| Means (SD) | 5.9 (2.9) | 5.8 (2.8) |
| Rhinitis duration |  |  |
| Missing | 250 (20.5) | 0 (0.0) |
| Intermittent | 648 (53.0) | 3,150 (68.7) |
| Persistent | 324 (26.5) | 1,434 (31.3) |
| Reported symptoms† |  |  |
| Rhinorrhoea |  |  |
| Missing | 412 (33.7) | 0 (0.0) |
| Never | 152 (12.4) | 1,191 (26.0) |
| Ever | 658 (53.9) | 3,393 (74.0) |
| Nasal congestion/obstruction |  |  |
| Missing | 565 (46.2) | 0 (0.0) |
| Never | 98 (8.0) | 1,143 (24.9) |
| Ever | 559 (45.7) | 3,441 (75.1) |
| Nasal itching |  |  |
| Missing | 662 (54.2) | 0 (0.0) |
| Never | 102 (8.4) | 1,597 (34.8) |
| Ever | 458 (37.5) | 2,987 (65.2) |
| Sneezing |  |  |
| Missing | 437 (35.8) | 0 (0.0) |
| Never | 101 (8.3) | 1,195 (26.1) |
| Ever | 684 (56.0) | 3,389 (73.9) |
| Associated-eye symptoms |  |  |
| Missing | 404 (33.1) | 36 (0.8) |
| Never | 166 (13.6) | 1,507 (32.9) |
| Ever | 652 (53.4) | 3,041 (66.3) |
| Number of symptoms |  |  |
| Missing | 1,035 (84.7) | 36 (0.8) |
| Means (SD) | 3.6 (1.3) | 3.5 (1.3) |
| Age of onset of rhinitis, year |  |  |
| Missing | 391 (32.0) | 975 (21.3) |
| Means (SD) | 28.8 (16.7) | 23.3 (14.8) |
| Reported triggers of symptoms* |  |  |
| Dust mites or house dust | 317 (25.9) | 1,620 (35.3) |
| Animals | 90 (7.4) | 587 (12.8) |
| Air pollution | 322 (26.4) | 1,238 (27.0) |
| Change in weather | 365 (29.9) | 1,312 (28.6) |
| Tobacco | 60 (4.9) | 299 (6.5) |
| Pollens | 551 (45.1) | 2,484 (54.2) |
| Cold air | 306 (25.0) | 1,117 (24.4) |
| Other | 154 (12.6) | 581 (12.7) |
| Unknown | 379 (31.0) | 1,194 (26.1) |
| Number of triggers |  |  |
| Missing | 0 (0.0) | 0 (0.0) |
| Means (SD) | 2.1 (1.2) | 2.3 (1.3) |
| Rhinitis treatment |  |  |
| Missing | 143 (11.7) | 54 (1.2) |
| Neither OA nor INCS | 406 (33.2) | 1,505 (32.8) |
| OA only | 189 (15.5) | 1,015 (22.1) |
| INCS only | 158 (12.9) | 542 (11.8) |
| OA and INCS | 326 (26.7) | 1,468 (32.0) |

Data are mean (SD) or n (%), BMI: Body Mass Index, INCS: Intranasal Corticosteroids, OA: Oral Antihistamines, TNSS4: Total Nasal Symptom Score 4, †: several possible answers.

Table S2: Multivariate logistic regressions comparing participants with ever asthma to those with never asthma stratified on ARIA classifications

|  | OR (95% CI) | | | |
| --- | --- | --- | --- | --- |
|  | Mild  Intermittent | Mild  Persistent | Mod-Sev Intermittent | Mod-Sev  Persistent |
| Reported symptoms |  |  |  |  |
| Rhinorrhoea | 1.16 (0.90-1.49) | 1.32 (0.84-2.09) | 1.14 (0.78-1.67) | 1.46 (0.91-2.32) |
| Nasal congestion/obstruction | 1.27 (0.99-1.64) | **2.05 (1.30-3.24)** | 0.91 (0.55-1.52) | 1.75 (0.80-3.82) |
| Nasal itching | **1.30 (1.00-1.68)** | 1.21 (0.80-1.83) | 1.01 (0.74-1.39) | **1.66 (1.08-2.55)** |
| Sneezing | 1.04 (0.80-1.35) | 1.26 (0.81-1.96) | **1.56 (1.07-2.29)** | **2.04 (1.23-3.38)** |
| Associated-eye symptoms | **1.52 (1.16-2.01)** | 0.80 (0.52-1.23) | 1.37 (0.96-1.95) | **1.75 (1.10-2.77)** |
| Reported triggers of symptoms |  |  |  |  |
| Dust mites or house dust | **3.62 (2.82-4.66)** | **2.37 (1.58-3.56)** | **2.02 (1.51-2.71)** | **3.39 (2.34-4.93)** |
| Animals | **3.26 (2.35-4.52)** | **3.21 (1.84-5.60)** | **3.20 (2.23-4.58)** | **2.44 (1.53-3.89)** |
| Air pollution | **1.50 (1.15-1.96)** | 1.02 (0.66-1.56) | 1.26 (0.93-1.71) | 1.19 (0.83-1.72) |
| Change in weather | 1.10 (0.84-1.44) | 1.07 (0.67-1.72) | 1.25 (0.93-1.68) | 1.17 (0.80-1.70) |
| Tobacco | **1.79 (1.09-2.96)** | **2.46 (1.27-4.75)** | **2.50 (1.53-4.09)** | 1.65 (0.91-2.99) |
| Pollens | 1.27 (0.99-1.63) | **1.73 (1.14-2.62)** | **1.54 (1.14-2.07)** | **2.08 (1.40-3.08)** |
| Cold air | 1.25 (0.95-1.64) | 0.83 (0.52-1.32) | 1.27 (0.92-1.74) | 1.40 (0.94-2.09) |
| Other | 0.67 (0.43-1.03) | **2.04 (1.21-3.44)** | 1.01 (0.67-1.51) | 1.39 (0.85-2.28) |
| Unknown | 0.46 (0.32-0.64) | 0.54 (0.34-0.86) | 0.57 (0.39-0.83) | 0.36 (0.23-0.57) |
| Rhinitis treatment |  |  |  |  |
| Neither OA nor INCS | ref. | ref. | ref. | ref. |
| OA only | **2.18 (1.58-3.02)** | **2.07 (1.13-3.77)** | **1.63 (1.07-2.48)** | **2.07 (1.01-4.27)** |
| INCS only | 1.20 (0.73-1.96) | 1.11 (0.51-2.39) | 0.85 (0.48-1.52) | 1.90 (0.85-4.22) |
| OA and INCS | **3.55 (2.59-4.86)** | **3.32 (1.94-5.69)** | **2.37 (1.61-3.48)** | **3.28 (1.71-6.29)** |
| Eosinophils count, cell/mm^3^  (per IQR increase) | **1.38 (1.19-1.59)** | **1.68 (1.31-2.14)** | **1.46 (1.23-1.74)** | **1.47 (1.24-1.74)** |
| Age of onset of rhinitis, year (per IQR increase) | **0.55 (0.45-0.67)** | **0.49 (0.35-0.68)** | **0.40 (0.30-0.53)** | **0.47 (0.34-0.65)** |
| TNSS4 (per IQR increase) | **1.42 (1.12-1.79)** | **1.71 (1.17-2.49)** | **1.19 (0.93-1.53)** | **1.56 (1.15-2.10)** |
| Number of symptoms (per IQR increase) | **1.35 (1.11-1.65)** | **1.36 (0.99-1.88)** | **1.29 (0.97-1.71)** | **2.03 (1.41-2.92)** |
| Number of triggers (per IQR increase) | **2.11 (1.72-2.59)** | **1.69 (1.27-2.25)** | **1.89 (1.53-2.35)** | **1.70 (1.35-2.16)** |

Logistic regression models adjusted for age, sex, smoking status, education, eczema and conjunctivitis status. Results are expressed as Odds Ratios (OR) with their Confidence Intervals (95% CI). For continuous quantitative variables, results are expressed for an increase of one interquartile range (IQR).Values in bold are those for which the p-value is < 0.05.
